# Central and Extrapontine Myelinolysis Affecting the Brain and Spinal Cord. An Unusual Presentation of Pancreatic Encephalopathy

**DOI:** 10.3389/fneur.2012.00135

**Published:** 2012-10-01

**Authors:** Alejandro Hornik, Federico J. Rodriguez Porcel, Caroline Agha, Murray Flaster, Sarkis Morales Vidal, Michael J. Schneck, John Lee, José Biller

**Affiliations:** ^1^Loyola University Medical CenterChicago, IL, USA

**Keywords:** pancreatic encephalopathy, osmotic myelinolysis, extrapontine myelinolysis, pontine myelinolysis, leukoencephalopathy

## Abstract

Pancreatic encephalopathy refers to a gamut of neuropsychiatric symptoms complicating acute pancreatitis. Osmotic myelinolysis is a known complication of pancreatic encephalopathy. We evaluated a 58-year-old woman with pancreatic encephalopathy associated to pontine and extrapontine myelinolysis involving the brain and spinal cord. To our knowledge, this is the first clinic pathological case report of pancreatic encephalopathy involving the spinal cord.

A 58-year-old right handed woman was brought to the emergency room after being found on the floor by her daughter. Fifteen days prior to admission she was admitted to an outside hospital for an ampulla of Vater adenoma resection and pancreatic duct stenting. An endoscopic retrograde cholangio-pancreatography (ERCP) was complicated by acute pancreatitis (serum lipase > 5000 units/L) due to stent migration. The stent was removed 2 days following the procedure. No electrolyte derangements were observed during her hospitalization. She remained hospitalized for another week and was reported to have “manipulative behavior.” It was also noted that 1 week prior to admission to our hospital (1 week post-surgery), she was diagnosed with depression and attention deficit disorder and was started on methylphenidate and bupropion. Her family stated she had become “less conversational, sleepy, and odd” and referred to a psychiatrist.

Medical history was remarkable for a chronic postprandial abdominal pain, hypertension, and a large calcified pelvic mass assumed to be a leiomyoma. Outpatient medications included lisinopril, tramadol, sustained release bupropion, and methylphenidate. Upon admission to our hospital, she complained of persistent abdominal pain and somnolence.

On examination, there was an apical systolic murmur, diffuse abdominal discomfort to palpation, normal bowel sounds, and no peritoneal signs.

Initial blood work showed a hemoglobin of 13.1 g/dL, white blood cell count (WBC) of 16.6 K/UL (lymphocytes 4%, monocytes 10%, granulocytes 85%), platelets 324 K/UL. Basic metabolic profile (BMP), liver enzymes, serum albumin, and billirubin were unremarkable. Serum amylase was 92 U/L and serum lipase was 31 U/L. Venous ammonia, urine drug screen, thyroid function tests, and troponins were unremarkable. Urinalysis showed moderate amounts of ketones and elevated urobilinogen. Activated partial thromboplastin time (aPTT) and International Normalized Ratio (INR) were normal. Cranial computed tomography (CCT) was unremarkable. Computed tomography (CT) of the chest, abdomen, and pelvis showed a 6 mm right hilar node, inflammatory changes of the pancreas consistent with pancreatitis, peripancreatic air, and abscess formation. There was also a 4 cm × 7 cm × 4 cm well defined soft tissue mass lesion with central calcification in the left lower quadrant of the abdomen (Figure 1). CT findings were remarkably improved compared to CT of the abdomen taken 3 weeks prior. General surgery consultant recommended intravenous meropenem. Transthoracic echocardiography (TTE) showed a left ventricular ejection fraction of 60%, moderate to severe aortic stenosis, and infero-lateral hypokinesis.

**Figure 1:**
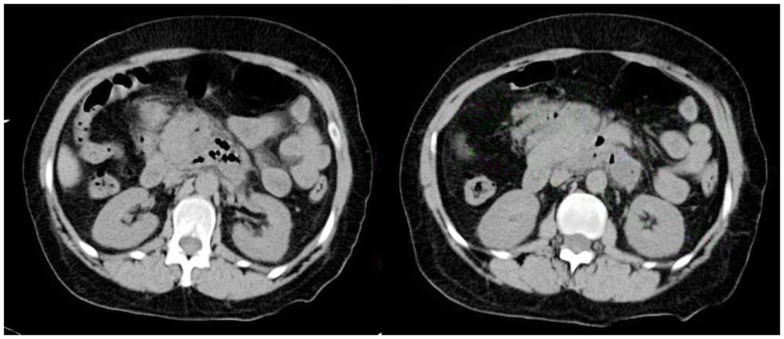
**CT of the abdomen shows an abnormal, poorly defined collection (6.9 cm × 4.7 cm) of gas and inflammatory changes in the region of the head and uncinate process of the pancreas and dorsal and inferior to the body of the pancreas**. A more focal (2.1 cm × 1.9 cm) fluid density along the posterior left aspect is noted.

Twelve hours following admission to our hospital, she became unarousable and was transferred to the Medical Intensive Care Unit (ICU). Neurology consultation was requested. Examination showed a comatose patient with a normal breathing pattern. She had symmetric facial grimacing to sternal rubbing. Pupils were 3 mm symmetric and reactive. Oculocephalic reflexes were normal. She had a flaccid areflexic tetraparesis, No extensor or flexor posturing were noted.

Magnetic resonance imaging (MRI) of the brain showed multiple areas of confluent restricted diffusion involving the splenium of the corpus callosum, right medial occipital white matter, right brachium pontis, bilateral dorsal pons, and right cerebral peduncle. These regions were associated with non-enhancing hyperintense T2 and fluid attenuated inversion recovery (FLAIR) sequence without mass effect (Figure 2).

**Figure 2:**
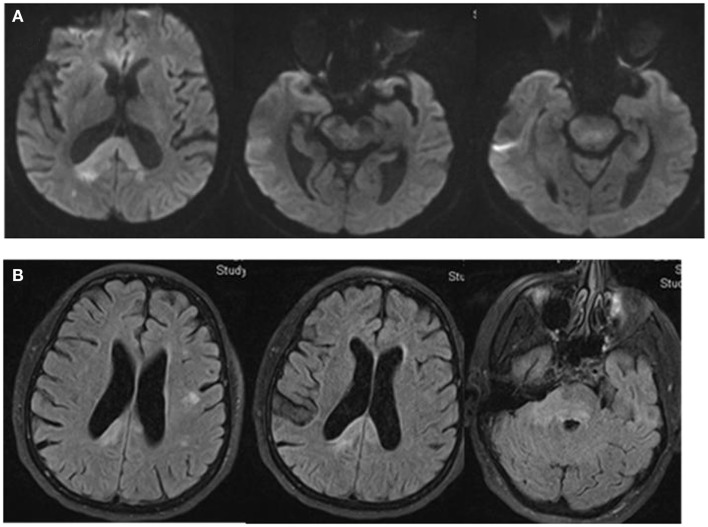
**DW-MRI of the brain shows multiple areas of confluent restricted diffusion affecting the splenium of the corpus callosum, right medial occipital white matter, right brachium pontis, bilateral dorsal pons, and right cerebral peduncle (A)**. These areas are hyperintense on Fluid Attenuated Inversion Recovery (FLAIR) sequence. There is no evidence of mass effect **(B)**.

A lumbar puncture (LP) showed an opening pressure of 19 cm of CSF. There were 176 RBC, 280 WBC (74% segmented, 2% lymphocytes, 10% monocytes), glucose content of 49 mg/dL (30% of serum glucose), and a protein content of 106 mg/dL. Smears, bacterial, viral, and fungal cultures, polymerase chain reaction (PCR) for Herpes Simplex Virus (HSV) type 1 and 2, flow cytometry, and cytology were negative.

The patient received intravenous Vancomycin 1 gram every 12 h, cefepime 2 g every 12 h, ampicillin 2 g every 4 h, metronidazole 500 mg every 6 h, and acyclovir 10 mg/kg every 12 h. Thirty-six hours after admission the patient exhibited anisocoria; the right pupil was 4 mm, sluggish and reactive; the left pupil was 2 mm and reactive. After elective intubation for airway protection, a repeat CCT was unchanged.

Repeat LP showed 106 RBC, 3 WBC (4% segmented, 86% lymphocytic, 10% macrophages), glucose content of 68 (45% of serum), and a protein content of 180. Smears, cultures, flow cytometry, and cytology again were negative. PCR for Epstein Bar virus (EBV), JC virus, cytomegalovirus (CMV), and cryptococal antigen were negative. Oligoclonal bands and myelin basic protein were negative.

Serial blood cultures failed to grow any bacteria. Serum human immunodeficiency virus (HIV) antibodies and HIV PCR analysis were negative. Serum paraneoplastic antibody panel (anti Hu, Ma1, Ma2, Yo, Ri, Car, Lems, CV2, Zix4, VGKC, Amphiphysin, G-ACHR), antiphospholipid antibody panel (beta 2 glycoprotein IgG, IgM, cardiolipin IgG, IgM), thyroid panel including thyroid stimulating hormone (TSH), free T4, T3, and thyroperoxidase (TPI) antibodies were all unremarkable.

The patient was transferred to the Neuroscience ICU (NICU). All antimicrobials were discontinued after 7 days of treatment. Repeat CT of the abdomen showed improvement of the pancreatic and peripancreatic abnormalities. Repeat MRI of the brain showed increased size, on diffusion weighted images, and T2/FLAIR, of the lesions located in the splenium, posterior white matter, midbrain, pons, and cerebellum. These lesions had faint enhancement following gadolinium administration (Figure 3). MRI of the cervical spine showed multiple non-contiguous spinal cord expansive lesions from the cervico-medullary junction to the level of the sixth cervical vertebra (Figure 4).

**Figure 3:**
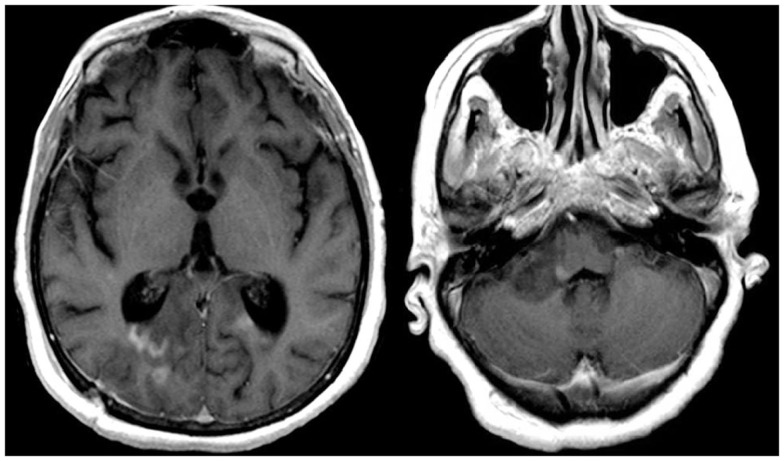
**T1W-MRI with contrast shows lesions located in the splenium, pons, and cerebellum with minimal contrast enhancement**.

**Figure 4:**
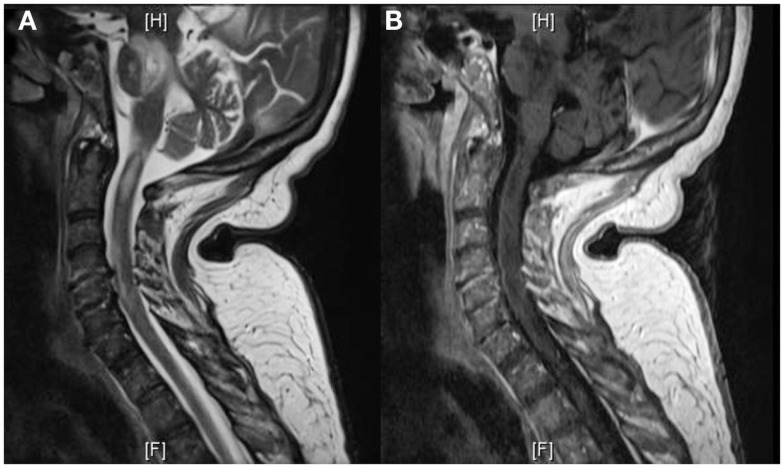
**T2W-MRI of the cervical spine shows multiple non-contiguous expansive lesions from the cervico-medullary junction to the level of the sixth cervical vertebra (A)**. T1W-MRI with contrast demonstrates patchy enhancement **(B)**.

Electroencephalography (EEG) showed moderate to severe diffuse background slowing consistent with a moderate to severe encephalopathy with a possible focal epileptiform activity arising from the right frontal region. The patient received 1500 mg of IV levetiracetam followed by 750 mg every 12 h with resolution of the ictal electrographic phenomenon. Somatosensory evoked potentials (SSEPs) of the upper and lower extremities were uninterpretable due to technical difficulties. Visual evoked potentials (VEP) showed integrity of the visual pathways.

A right burr hole stereotactic brain biopsy of the splenium and right occipital lobe showed reactive gliosis and numerous macrophages. Specific stains for differentiation CD20 (B-cell) and CD3 (T-cell) showed no prominent lymphocytic infiltrates. Luxol Fast Blue and Bielschowsky stains showed evidence of demyelination, with relative preservation of axons. Differential diagnosis at this point included pontine and extrapontine myelinolysis (EPM). Despite the scant presence of perivascular lymphocytic inflammatory infiltrates, a diagnosis of possible acute disseminated encephalomyelitis (ADEM) was also entertained.

The patient received1500 mg of IV methylprednisolone daily for 5 days followed by plasmapheresis for a total of five plasma exchanges. However, she had no clinical improvement. The patient remained hospitalized in critical condition for 3 weeks. Repeat brain and cervical spine MRIs showed no improvement. Hospital course was complicated by ventilator associated pneumonia and hypotension requiring vasopressors. Based on advanced directives, the family decided to discontinue vital support 5 weeks after initial admission to our hospital. She suffered a respiratory arrest 4 days after extubation resulting in her death.

## Autopsy Findings

**Figure 5:**
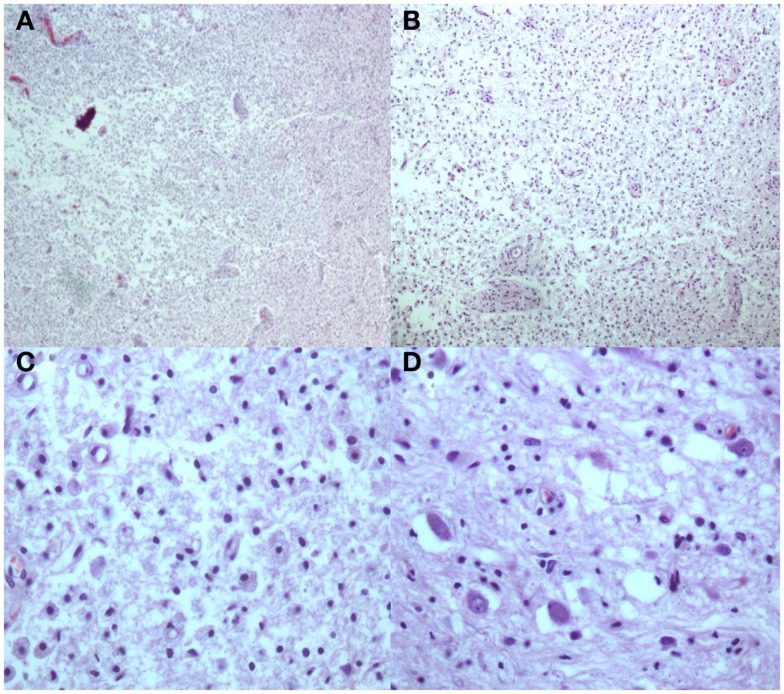
**H and E stains [(A,B) Low Power; (C) High Power] Multiple white matter areas containing myelin loss and numerous macrophages with focal cavitation**. Adjacent to areas of myelin loss there is relative preservation of neurons [**(D)** High Power].

**Figure 6:**
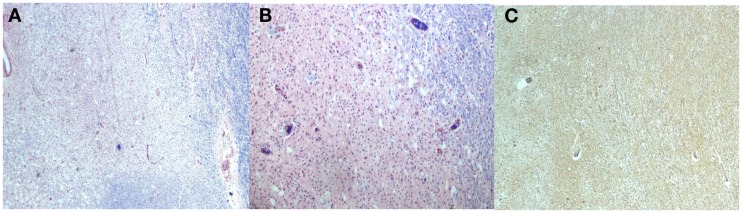
**Luxol Fast Blue stains [(A,B) Low power] shows areas with loss of myelin and increased macrophages**. The neurofilament stain [**(C)**, Low power] with preservation of the axons primarily at the periphery of the lesions.

Macroscopic analysis showed thin translucent leptomeninges without subarachnoid hemorrhage. There were multiple distinct white matter lesions including the right occipital lobe, splenium, and right middle cerebellar peduncle extending into the central pons. These multiple white matter areas contained numerous macrophages with focal cavitation containing scattered perivascular and parenchymal T cells (CD3) and B-cells (CD20). The neurofilament stains revealed preservation of the axons at the periphery of the lesions, but there was axonal loss in the middle of the cavitary portions of some of the lesions. Luxol Fast Blue myelin stains showed areas of demyelination including portions of the medullary medial lemniscus. Also noted were hyalinized barrel-shaped vessels in the parietal and occipital cerebral cortical white matter as well as in the basal ganglia and amygdale consistent with mild arteriolar sclerosis. Focal gliosis and microglial proliferation in the thalamus was noted. A simian virus antibody 40 (SV40) stain for progressive multifocal leukoencephalopathy (polyoma virus) was negative. In summary, there were multiple cavitated pontine and extrapontine myelinolytic type lesions with extensive macrophages, focal cavitation, and scant inflammatory infiltrates in the setting of peripheral pancreatic autolytic changes and peripancreatic fat necrosis and chronic inflammation consistent with pancreatic encephalopathy (Figures 5 and 6).

## Discussion

The osmotic myelinolytic syndromes combine signs and symptoms of central nervous system (CNS) myelinolysis. These disorders either affect the pons resulting in central pontine myelinolysis (CPM), or other CNS resulting in EPM.

Typical symptoms of CPM are biphasic (Adams et al., 1959). Initially, presenting with encephalopathy or seizures due to hyponatremia. As normonatremia is established, patients recover to subsequently deteriorate days later with a flaccid and then spastic quadriparesis, dysphagia, and dysarthria. If the myelinolytic process extends to the tegmentum, pupillary, and oculomotor abnormalities are noted.

Extrapontine myelinolysis may affect multiple areas within the CNS (Table 1). CPM and EPM may occur in isolation or combined (Gotch and Calmant, 1987) as in our patient. The coexistence of CPM and EPM may account for a protean clinical picture, often preceded by a variety of psychiatric manifestations. The underlying pathology of these lesions is characterized by degeneration and loss of oligodendrocytes with preservation of axons. Lesions are sharply demarcated. During the active phase of the disease, they contain sheets of lipid-laden macrophages and large number of reactive astrocytes. Infiltration by lymphocytes is sparse or absent (Love, 2006). A variety of potential etiologies have been identified (Table 2; Tomlinson et al., 1976; Martin, 2004). Patients with osmotic myelinolysis also have a high prevalence of concomitant hypokalemia (Lohr, 1994). Myelinolysis is best appreciated on MRI, presenting with non-enhancing hyperintense lesions on T2 and hypointense lesions on T1.

**Table 1:** **CNS lesions on EPM**.

Cerebellum
Lateral geniculate body
External capsule
Extreme capsule
Hippocampus
Putamen
Subcortical areas
Thalamus
Caudate nucleus
Claustrum
Internal capsule
Midbrain
Internal medullary lamellae
Mamillary bodies
Medulla oblongata

**Table 2:** **Disorders associated with myelinolysis**.

Alcoholism
Malnutrition
Prolonged diuretic use
Psychogenic polydipsia
Burns
Post liver transplant
Post pituitary surgery
Post urological/gynecological surgery (s/p glycine infusion)
Pancreatitis

Pancreatic encephalopathy (Rothermich and Von Haam, 1941), refers to a variety of neuropsychiatric symptoms complicating acute pancreatitis (Jacewicz and Marino, 2007). Encephalopathy has been reported in 9–35% of subjects suffering acute pancreatitis without history of alcoholism (de Falco et al., 1980). Neurological manifestations related to acute pancreatitis are multifactorial and may result from hypocalcemia, hypomagnesemia, low thiamine levels, or osmotic myelinolysis. Symptoms due to osmotic myelinolysis include but are not limited to fluctuating mental status, disorientation, confusion, dysarthria, hallucinations, delirium, akinetic mutism, seizures, and coma (Sharf and Bental, 1971). Symptoms usually present 2–5 days after onset (Chan et al., 2003), although new neurological symptoms have been reported more than 1 month after onset of pancreatitis (Ding et al., 2004). CNS pathological changes include patchy myelin pallor (Vogel, 1951), CPM, EPM (Sherins and Verity, 1968), acute hemorrhagic leukoencephalitis (Chan et al., 2003), and fat embolism (Bhalla et al., 2003). Vogel first described scattered foci of intense demyelinization in cases of pancreatic encephalopathy. He was able to demonstrate similar pathological effect in animals by injecting lipase into the CNS (Vogel, 1951). Other putative physiopathologic explanations have been postulated including pancreatin activation, cytokines such as tumor necrosis factor alpha (TNF-α), Interleukin 1(IL 1). These pro-inflammatory markers and pancreatic enzymes were accounted to increase blood brain barrier permeability, causing vasogenic edema, myelinolysis, inflammatory activation, electrolytic disturbances, and hyperosmolarity due to osmotic diuresis (Zhang, 2007). Animal models of acute pancreatitis have subsequently proven the effect of the mentioned markers (Farkas et al., 1998). Differential diagnosis of altered mental status among patients with pancreatitis is extensive. Pancreatic encephalopathy should only be considered after more likely possibilities including ischemia, uremia, hypoxemia, electrolyte abnormalities, thiamine deficiency, have been excluded (Jacewicz and Marino, 2007). CSF analysis on patients with pancreatic encephalopathy show high protein content, mild lymphocytosis, and lipase (Vogel, 1950; Estrada et al., 1979; Sjaastad et al., 1979).

There is no known treatment for the osmotic demyelinating syndrome regardless of etiology. In cases of pancreatic encephalopathy, specific anti-enzymes, such as aprotinin (Trasylol^®^), have been proposed. This is a low molecular weight polypeptide produced by the parotid glands and lung tissues. Aprotinin inhibits the action of trypsin, chymotrypsin, kallikrein, fibrinolysin, and other proteolytic enzymes (Sharf and Levy, 1976). Other proposed alternatives for the treatment of myelinolysis include reinduction of hyponatremia (Oya et al., 2001), recombinant human growth hormone (Qian et al., 2001), plasmapheresis, IVIG, and corticosteroids (Kumar et al., 2006).

## Summary

Our patient had pathology proven pancreatic encephalopathy affecting large areas of the white matter throughout brain, brainstem, and spinal cord. The cystic cavitating components of our patient’s lesions most likely could be explained by direct proteolytic effect of pancreatic enzymes, including lipase. To our knowledge, there is only one other report of a patient presenting with osmotic myelinolysis affecting the spinal cord, but without accompanying and supporting autopsy pathology findings (Pneumatikos et al., 1998).

## Conflict of Interest Statement

The authors declare that the research was conducted in the absence of any commercial or financial relationships that could be construed as a potential conflict of interest.
